# Implementation of guidelines about women with previous cesarean section through educational/motivational interventions

**DOI:** 10.1002/ijgo.14212

**Published:** 2022-04-22

**Authors:** Francesca Monari, Daniela Menichini, Emma Bertucci, Isabella Neri, Enrica Perrone, Fabio Facchinetti

**Affiliations:** ^1^ Obstetrics Unit, Department of Medical and Surgical Sciences for Mother, Child and Adult University of Modena and Reggio Emilia. University Hospital Policlinico of Modena, Italy Modena Italy; ^2^ International Doctorate School in Clinical and Experimental Medicine, Department of Biomedical, Metabolic and Neural Sciences University of Modena and Reggio Emilia Modena Italy; ^3^ Department of Biomedical and Neuromotor Sciences (DIBINEM), Hygiene and Preventive Medicine Section, Alma Mater Studiorum University of Bologna Bologna Italy

**Keywords:** Grobman score, health quality improvement, implementation, motivational intervention, Neonatal outcomes, professional attitudes, reduction, repeat cesarean section, Robson group, vaginal birth after cesarean section

## Abstract

**Objective:**

To investigate the effect of a quality improvement project with an educational/motivational intervention in northern Italy on the implementation of the trial of labor after cesarean section (CS).

**Method:**

A pre‐post study design was used. Every birth center (*n* = 23) of the Emilia‐Romagna region was included. Gynecologist opinion leaders were first trained about Italian CS recommendations. Barriers to implementation were discussed and shared. Educational/motivational interventions were implemented. Data of multipara with previous CS, with a single, cephalic pregnancy at term, were collected during two periods, before (2012–2014) and after (2017–2019) the intervention (2015–2016). The primary outcome was the rate of vaginal birth after CS (VBAC) and perinatal outcomes.

**Results:**

A total of 20 496 women were included. The VBAC rate increased from 18.1% to 23.1% after intervention (*P* < 0.001). The likelihood of VBAC—adjusted for age 40 years or older, Caucasian, body mass index (BMI, calculated as weight in kilograms divided by the square of height in meters) at least 30, previous vaginal delivery, and labor induction—was increased by the intervention by 42% (odds ratio 1.42, 95% confidence interval 1.31–1.54). Neonatal well‐being was improved by intervention; neonates requiring resuscitation decreased from 2.1% to 1.6% (*P* = 0.001).

**Conclusion:**

Educating and motivating gynecologists toward the trial of labor after CS is worth pursuing. Health quality improvement is demonstrated by increased VBAC even improving neonatal well‐being.

## Synopsis

Educating gynecologists toward trial of labor after cesarean section is an intervention that leads to increasing vaginal birth after cesarean section, even improving neonatal well‐being.

## 1 INTRODUCTION

Cesarean section (CS) is an effective and life‐saving obstetric intervention in the presence of conditions complicating pregnancy or labor. However, it is associated with both immediate and long‐term maternal and perinatal risks.^1^, ^2^ Rates of CS have been increasing everywhere in the past decades,^3^ becoming a pervasive phenomenon. This constant rise^4^ is a public health concern in developed countries despite large variation among them.^5^ Major factors contributing to the increase in CS rates include primary CS, the increase of labor inductions, and the decrease in vaginal birth after CS (VBAC).

Elective repeat CS (RCS) and VBAC for women with a previous CS are both associated with benefits and harms.^6^ Most studies report an increase in adverse maternal and neonatal outcomes following RCS.^7^, ^8^ Hence, the approach of the trial of labor after CS (TOLAC) provides the opportunity to achieve a VBAC for women with a history of one or two previous low‐transverse incisions, in the absence of further risk factors.^9^ This advice was promoted by several organizations including the American College of Obstetricians and Gynecologists in 2010,^10^ the National Institute for Health and Care Excellence in 2013,^11^ and the Italian Superior Health Institute, which released guidelines in 2012.^12^ Despite the evidence of safety and feasibility of TOLAC and the recognized health benefits of vaginal birth, the average rate of VBAC in Italy did not change, remaining lower than 10%^13^ in recent years.

As Italy has one of the highest CS rates (31.1%) in Europe,^14^ and considering that northern European countries (Sweden, Finland) have reached a 45%–55% VBAC rate, there is an urgent need to develop and evaluate multifaceted prenatal and perinatal interventions to effectively reduce the number of unnecessary CS in Italy, also promoting VBAC where appropriate.^12^, ^15^

Few studies have evaluated the effects of clinician‐centered interventions to promote VBAC, and the available data show conflicting results.^16^, ^17^ A systematic review on this topic reported that the only strategy that significantly increased VBAC rates was an educational intervention provided by an opinion leader.^15^ Moreover, interventions such as audit feedback, quality improvement, and multifaceted strategies are effective ways to change clinical practice and reduce the rate of CS.^18^ Finally, a recent multicenter cluster trial—the QUARISMA trial—involving more than 180 000 participants showed that a multifaceted intervention reduced the risk of CS in low‐risk pregnancies.^19^

On these bases, we decided to perform an area‐based quality improvement program focusing on the implementation of Italian guidelines related to VBAC in Emilia‐Romagna, a north Italian region, characterized by a proactive policy of health quality.

## 2 MATERIALS AND METHODS

### 2.1 Study population

The present pre‐post study is part of a project endorsed by the Regional Health Authority of Emilia‐Romagna, a regional governmental body accountable for issuing routinely collected anonymized patient data to research institutions. Institutional Review Board or Ethics Committee approval was not needed because, according to the Italian privacy law (Legislative Decree 101/2018, D. Lgs 101/2018), regional National Health Service data can be used for scientific purposes provided sensitive information is anonymized.

Emilia‐Romagna is a region of central Italy that accounts for about 30 000 deliveries/year occurring in 23 public birth centers. Data about birth certificates have been issued annually since 2003 and analyzed in a public report.^20^

The study included every woman categorized in Robson Class V^21^ who delivered in Emilia‐Romagna in the period 2012–2019.

### 2.2 Intervention

Gynecologist opinion leaders were first trained in Italian National Health Service recommendations on the appropriate use of CS, counseling on VBAC, and knowledge of Grobman score. The gynecologist opinion leader was the most experienced as well as the most motivated physician with attitudes in counseling on the mode of delivery. The training was performed for one opinion leader for each birth unit. Barriers to implementation and possible solutions were discussed locally and shared at the regional level. The educational/motivational interventions program included: (1) training of physicians regarding evidence‐based clinical practices conducted by gynecologist opinion leaders, (2) implementation of clinical audits in each birth unit by a multidisciplinary perinatal team, and (3) implementation of best practices in each birth unit and joint discussions by women and clinicians.

Moreover, distance technology‐assisted learning resources were also developed to implement professionals' knowledge and counseling attitudes. In each birth unit, the local intervention identified was implemented for 2 years, during 2015 and 2016.

Data of multipara with one or two previous CS, with a single, cephalic pregnancy at term, were captured from birth certificates in two periods, before (2012–2014) and after (2017–2019) the intervention, which occurred in 2015–2016.

The primary outcome was the rate of VBAC in multiparous women with at least one previous CS, with a single cephalic pregnancy, at 37 weeks or more of pregnancy (Robson group 5), comparing the pre‐intervention (2012–2014) and post‐intervention (2017–2019) periods. According to the Grobman nomogram, the main outcome was adjusted for known maternal conditions affecting VBAC success, i.e., previous vaginal delivery, pre‐pregnancy body mass index (BMI; calculated as weight in kilograms divided by the square of height in meters) classes, maternal age 40 years or older, maternal education level, and place of origin (Italian or foreign). Educational/motivational VBAC intervention was also included. Relevant perinatal outcomes were collected.

### 2.3 Data sources

Data from the 23 maternity services of Emilia‐Romagna during calendar years 2012–2014 and 2017–2019 were extracted from the Emilia‐Romagna Birth Certificates (CedAP is the Italian acronym), including the mother's sociodemographic information (maternal age, education, place of origin, smoking, occupation, BMI), obstetric history (previous delivery, vaginal delivery and assisted reproductive technologies), clinical information on the current pregnancy (antenatal course, mode of labor and delivery, gestational age at birth), and the newborn (birth weight, stillbirth, Apgar score < 7, need for resuscitation) collected within 10 days of delivery by the attending midwife of all Maternity units.

### 2.4 Statistical methods

Analyses were set up by comparing the two study groups “before” and “after” the educational/motivational intervention. Student *t* test and χ^2^ test were performed for continuous and categorical variables, respectively. Continuous variables were described as the mean ± standard deviation (SD), whereas categorical variables were described as the absolute and percentage frequencies. The multivariable prediction model for the risk of having an RCS was developed by carrying out the following steps. First, univariate logistic regression models were used to assess the relationship among each relevant independent variable. The final prediction model was determined by a stepwise backward selection procedure in which only independent variables associated with RCS risk with *P* value less than 0.05 were retained. Results of logistic models were reported as the odds ratio (OR) with 95% confidence interval (CI) and Wald *P* value. Statistical analyses were performed with stata 16.1 (StataCorp. 2019).

## 3 RESULTS

During the study period a total of 269 497 women delivered in the area: 106 014 of them gave birth in the pre‐intervention period (2012–2014) while 95 813 women delivered during the post‐intervention period (2017–2019), including 11 035 (10.4%) and 9461 (9.8%) women (total 20 496) classified in Robson group 5, respectively. The post‐intervention was smaller, in line with the overall reduction of the births from 2012 to 2019.

The characteristics of the population are reported in Table 1, divided between the pre‐intervention and post‐intervention groups. Maternal age was slightly higher in the post‐intervention group (Pre‐intervention 33.7 years vs. Post‐intervention 33.9 years; *P* = 0.001) whereas a significantly lower rate of Italian women was found in the post‐intervention group (Pre‐intervention 66.2% vs. Post‐intervention 58.3%; *P* < 0.001). Similarly, obese women and women conceiving with assisted reproductive technologies significantly increased in the post‐intervention period.

**TABLE 1:** Maternal baseline characteristics^a^

Characteristics	Pre‐intervention (*N* = 11 035)	Post‐intervention (*N* = 9461)	*P* value
Mean maternal age, years	33.7 ± 4.9	33.9 ± 5.0	0.001
Maternal age ≥ 40 years	1296 (11.7)	1273 (13.5)	0.001
Maternal education level			0.038
High	2976 (27.0)	2658 (28.1)	
Medium	4527 (41.0)	3720 (39.3)	
Low	3532 (32.0)	3083 (32.6)	
Italian place of origin	7310 (66.2)	5518 (58.3)	0.001
Smoking habits	669 (6.1)	520 (5.6)	0.001
Occupation			0.001
Employed	539 (8.0)	409 (7.9)	
Unemployed	356 (5.3)	206 (4.0)	
Looking for the first occupation	174 (2.6)	159 (3.1)	
Student	3484 (51.5)	2554 (49.4)	
Housewife	1622 (24.0)	1246 (24.1)	
Other condition	589 (8.7)	597 (11.5)	
BMI classes			0.001
Underweight	495 (7.4)	598 (6.3)	
Normal weight	3797 (56.5)	4992 (52.8)	
Overweight	1576 (23.5)	2448 (25.9)	
Obese	850 (12.6)	1423 (15.0)	
Previous deliveries			0.001
1	8374 (75.9)	6742 (71.3)	
2	2265 (20.5)	2261 (23.9)	
3	292 (2.6)	340 (3.6)	
≥ 4	104 (0.9)	117 (1.2)	
Previous vaginal deliveries			0.001
0	9911 (89.8)	8241 (87.1)	
1	828 (7.5)	881 (9.3)	
>1	291 (2.6)	339 (3.5)	
Assisted reproductive technology	78 (0.7)	90 (1.0)	0.05

Abbreviation: BMI, body mass index (calculated as weight in kilograms divided by the square of height in meters).

^a^ Data are presented as mean ± standard deviation or as number (percentage).

Compared with the pre‐intervention group, the post‐intervention group presented an overall significant reduction in RCS rate (Pre‐intervention 9043, 81.9% vs. Post‐intervention 7278, 76.9%; *P* < 0.001), corresponding to a 5.0% reduction (Table 2). Indeed, we found that the rate of elective CS was significantly lower, whereas the rate of induction of labor in the post‐intervention group was higher (Pre‐intervention 3.3% vs. Post‐intervention 6.4%; *P* < 0.001), indicating different management of women with previous CS in the latter group, accompanied by a significantly higher rate of women delivering between 39 and 41 weeks of pregnancy (*P* = 0.001). Table S1 in the supplementary material shows the different distribution of VBAC pre‐ and post‐intervention across the 23 hospitals that participated in the study.

**TABLE 2:** Pregnancy outcomes^a^

Outcomes	Pre‐intervention (*N* = 11 035)	Post‐intervention (*N* = 9461)	*P* value
Antenatal course			0.001
No, attended in previous pregnancy	2449 (22.3)	2381 (25.5)	
No	7877 (71.8)	6271 (67.0)	
Yes, at a public family clinic	452 (4.1)	522 (5.6)	
Yes, at a public hospital	131 (1.2)	94 (1.0)	
Yes, in a private facility	55 (0.5)	88 (0.9)	
Mode of labor			0.001
Spontaneous onset labor	2652 (24.0)	2351 (24.9)	
Induced labor	363 (3.3)	603 (6.4)	
Absent	8020 (72.7)	6507 (68.8)	
Mode of delivery			<0.001
Spontaneous vaginal delivery	1797 (16.3)	2013 (21.3)	
Forceps extraction	6 (0.1)	6 (0.1)	
Vacuum extraction	189 (1.7)	164 (1.7)	
Emergency cesarean section	1722 (15.6)	1583 (16.7)	
Elective cesarean section	7321 (66.3)	5695 (60.2)	
Vaginal delivery	1992 (18.0)	2183 (23.1)	0.001
Elective cesarean section	7321 (81.0)	5695 (78.2)	0.001
Cesarean section			0.001
In labor	978 (10.8)	641 (8.8)	
Without labor	8065 (89.2)	6637 (91.2)	
Mean gestational age at birth, weeks	38.7 ± 1.0	38.8 ± 1.0	0.001
Gestational age			0.001
37	10.35 (9.4)	811 (8.6)	
38	4273 (38.7)	3061 (32.3)	
39	3676 (33.3)	3564 (37.7)	
40	1391 (12.6)	1371 (14.5)	
41+	660 (6.0)	654 (6.9)	

^a^ Data are presented as mean ± standard deviation or as number (percentage).

There were no significant differences for neonatal outcomes, besides the rate of large‐for‐gestational‐age neonates, which was higher in the pre‐intervention group (Pre‐intervention 12.7% vs. Post‐intervention 11.4%; *P* = 0.005) (Table 3). Neonatal well‐being was improved by intervention; number of neonates requiring resuscitation decreased from 232 (2.1%) to 156 (1.6%) (*P* = 0.001), requiring less heart massage, adrenaline, and other drugs (*P* < 0.02) (Table 4).

**TABLE 3:** Neonatal outcomes^a^

Outcomes	Pre‐intervention (N = 11 035)	Post‐intervention (N = 9461)	*P* value
Mean birth weight, g	3336.9 ± 431.5	3326.1 ± 432.7	0.07
Macrosomia (>4000 g)	730 (6.6)	597 (6.3)	0.37
Small for gestational age	1083 (9.8)	977 (10.3)	0.22
Large for gestational age	1400 (12.7)	1078 (11.4)	0.005
Stillbirth	9 (0.1)	11 (0.1)	0.43
Stillbirth	6 (0.0)	8 (0.1)	0.58
Before labor	0 (0.0)	1 (0.0)	
During childbirth (expulsive period)	3 (0.0)	2 (0.0)	
Time of death unknown			
Apgar score ≤7	101 (0.9)	87 (0.9)	0.97
Need for resuscitation	232 (2.1)	156 (1.6)	0.001

^a^ Data are presented as mean ± standard deviation or as number (percentage).

**TABLE 4:** Resuscitation details^a^

Resuscitation	Pre‐intervention (*N* = 11 035)	Post‐intervention (*N* = 9461)	*P* value
Manual ventilation	186 (0.017)	148 (0.016)	0.24
Intubation	40 (0.004)	31 (0.003)	0.33
Heart massage	26 (0.002)	10 (0.001)	0.01
Adrenaline	28 (0.003)	6 (0.001)	0.001
Other drug	19 (0.002)	7 (0.001)	0.02

^a^ Data are presented as number (percentage).

The results of the multivariable analyses are reported in Table 5. The variables impacting on the likelihood of having a VBAC were: a previous vaginal delivery (OR 1.5, 95% CI 1.4–1.6; *P* = 0.000), the educational/motivational intervention (OR 1.45, 95% CI 1.3–1.6; *P* < 0.001), the pre‐pregnancy BMI classes (OR 0.93, 95% CI 0.8–0.9; *P* = 0.003), and maternal age 40 years or older (OR 0.84, 95% CI 0.7–0.9, *P* = 0.008) (Table 5).

**TABLE 5:** Multivariable analysis for the likelihood of having a vaginal delivery

	OR	95% CI	*P* value
Previous vaginal deliveries	1.50	1.41–1.61	0.001
VBAC intervention	1.45	1.35–1.58	0.001
BMI classes	0.93	0.88–0.97	0.003
Maternal age ≥ 40 years	0.84	0.75–0.96	0.008
Maternal education level	1.00	0.95–1.06	0.92
Italian place of origin	0.98	0.90–1.06	0.63

Abbreviations: BMI, body mass index (calculated as weight in kilograms divided by the square of height in meters); CI, confidence interval; OR, odds ratio; VBAC, vaginal birth after cesarean section.

The educational/motivational intervention (OR 1.45, 95% CI 1.3–1.6; *P* < 0.001), adjusted for potential risk factors, was associated with having a vaginal delivery.

## 4 DISCUSSION

The educational motivational intervention implemented for 2 years in our region allowed an overall increase in VBAC rate of around 5%, although with heterogeneity among birth centers.

Such an effect has been obtained with a multidisciplinary program, including audits regarding the indications for CS, feedback to health professionals, and implementation of best practices. Few studies have evaluated similar programs. A systematic review evaluated the effects of clinician‐centered interventions.^15^ Three studies were included, one of them evaluating a leader educational strategy, which has shown benefits for increasing VBAC rates. However, all those studies were performed before 1996. In contrast, recent studies demonstrated that neither the use of decision aids nor the education of women has a significant effect on VBAC rates.^22^, ^23^ On the other hand, a multinational randomized controlled trial including Italy on 2002 women from 15 different maternity centers, showed a large increase of the VBAC rate from 8% to 22%.^17^ They implemented interventions focused on the use of opinion leaders (one midwife and one obstetrician per birth unit), education and support to women as well as healthcare professionals, and discussion between physicians and women, to reach a shared decision on delivery mode. This agrees with our findings and demonstrates that a multifaceted intervention could be a successful strategy.

It must be underlined that risk factors for unsuccessful VBAC, as reported in the Grobman nomogram, were significantly increased in the population during the post‐intervention period. Nevertheless, the educational/motivational program was effective in reducing the CS rate in women of Robson group 5.^24^

A limitation of our study is the lack of data about uterine ruptures occurring in the different periods. However, the safety of increasing VBAC was witnessed by the stable rate of neonatal complications requiring intensive care admission. A positive effect of having reduced RCS was demonstrated by the lower rate of neonates needing resuscitation in the post‐intervention period, confirming safe and good outcomes described in successful VBAC.^7^ This finding might depend on the reduction of births below 39 weeks of pregnancy when a residual number of neonates born through CS still develop respiratory distress syndrome.^8^

Another limitation of this study is the differential distribution of VBAC across the 23 hospitals included in the study, suggesting heterogeneity in the implementation of the program. These data were reported in Table S1. This could depend on several factors including leadership and the well‐known negative attitude of change in clinical practice, especially among older providers.

The quality improvement program of our study, in line with the recently published one,^25^ includes an educational intervention to transfer evidence‐based practice provided by an opinion leader, local clinical audits, feedback, and implementation of best practices. This program should be evaluated as a protocol to introduce and enforce at the hospital level to contain the number of RCS and promote VBAC where appropriate. All eligible women should be offered the option of TOLAC as a standard policy, especially in centers equipped with an anesthesiology unit dedicated to the labor ward, a blood bank, and an interventional radiology unit. These supports allow clinicians appropriate management of the dramatic, though rare, emergencies of uterine rupture. Although this intervention approach seems to be effective, further research to improve the best way of promoting VBAC is essential.

In conclusion, educating and motivating gynecologists toward TOLAC is worth pursuing. Health quality improvement is demonstrated by increased VBAC, even improving neonatal well‐being.

## ACKNOWLEDGMENTS

Open Access Funding provided by Universita degli Studi di Modena e Reggio Emilia within the CRUI‐CARE Agreement.

[Correction added on 08‐May‐2022, after first online publication: CRUI‐CARE funding statement has been added.]

## CONFLICTS OF INTEREST

The authors have no conflict of interests.

## AUTHOR CONTRIBUTIONS

FM, FF, and DM made substantial contributions to the conception or design of the work, the acquisition, analysis, interpretation of data for the work, and wrote the paper; FM, DM, EP, EB, and IN drafted the work or revised it critically for important intellectual content. FF gave final approval of the version to be published.

## Supporting information


Table S1

## Data Availability

Data derived from public domain resources
