# Supplementary material for: Implementation of guidelines about women with previous cesarean section through educational/motivational interventions
Source: Int J Gynaecol Obstet. 2022 Apr 22;159(3):810–6. doi: 10.1002/ijgo.14212 (PMC9790249; doi:10.1002/ijgo.14212)
Supplement: Supplementary file 1 — Table S1 [file IJGO-159-810-s001.docx]

**Supplementary Materials**

**Table. Differential distribution of VBAC in Class V group across the Emilia Romagna hospitals**

|  | **Pre-Intervention**  **N=11035** | | | **Post-Intervention**  **N=9461** | | | **Delta %** | **p value** |
| --- | --- | --- | --- | --- | --- | --- | --- | --- |
|  | **N of CS** | **N of total deliveries of Class V** | **%** | **N of CS** | **N of total deliveries of Class V** | **%** |  |  |
| **A.** | 1022 | 1264 | 80.85 | 682 | 744 | 91.67 | 10.81 | **<0.001** |
| **B.** | 877 | 1159 | 75.67 | 553 | 850 | 65.06 | -10.61 | **<0.001** |
| **C.** | 721 | 908 | 79.41 | 689 | 882 | 78.12 | -1.29 | 0.25 |
| **D.** | 595 | 949 | 62.70 | 373 | 675 | 55.26 | -7.44 | **<0.001** |
| **E.** | 694 | 777 | 89.32 | 540 | 785 | 68.79 | -20.53 | **<0.001** |
| **F.** | 525 | 616 | 85.23 | 509 | 615 | 82.76 | -2.46 | **<0.001** |
| **G.** | 381 | 415 | 91.81 | 430 | 516 | 83.33 | -8.47 | **<0.001** |
| **H.** | 456 | 564 | 80.85 | 348 | 543 | 64.09 | -16.76 | **<0.001** |
| **I.** | 394 | 417 | 94.48 | 476 | 575 | 82.78 | -11.70 | **<0.001** |
| **a.** | 532 | 589 | 90.32 | 597 | 671 | 88.97 | -1.35 | 0.21 |
| **b.** | 432 | 495 | 87.27 | 278 | 403 | 68.98 | -18.29 | **<0.001** |
| **c.** | 228 | 357 | 63.87 | 184 | 310 | 59.35 | -4.51 | 0.11 |
| **d.** | 308 | 327 | 94.19 | 351 | 394 | 89.09 | -5.10 | **0.007** |
| **e. F** | 287 | 311 | 92.28 | 244 | 340 | 71.76 | -20.52 | **<0.001** |
| **f.** | 212 | 215 | 98.60 | 224 | 230 | 97.39 | -1.21 | 0.18 |
| **g.** | 245 | 251 | 97.61 | 208 | 233 | 89.27 | -8.34 | **<0.001** |
| **h.** | 185 | 252 | 73.41 | 38 | 46 | 82.61 | 9.20 | 0.09 |
| **i.** | 175 | 234 | 74.79 | 23 | 29 | 79.31 | 4.52 | 0.28 |
| **l.** | 184 | 233 | 78.97 | 142 | 147 | 96.60 | 17.63 | **<0.001** |
| **m.** | 208 | 230 | 90.43 | 98 | 102 | 96.08 | 5.64 | **0.04** |
| **n.** | 159 | 182 | 87.36 | 108 | 143 | 75.52 | -11.84 | **0.002** |
| **o.** | 128 | 159 | 80.50 | 112 | 129 | 86.82 | 6.32 | 0.07 |
| **p.** | 95 | 131 | 72.52 | 71 | 99 | 71.72 | -0.80 | 0.44 |

*A-I: provide obstetric and neonatal intensive care (Hub)*

*a-p: provide obstetric and neonatal care, except for the sickest babies (there is not neonatal intensive care unit)*
